# Comparison of cervical muscle activity and spinal curvatures in the sitting position with 3 different sloping seats

**DOI:** 10.1097/MD.0000000000021178

**Published:** 2020-07-10

**Authors:** JongEun Yim, Junhyuck Park, Everett Lohman, KwangSun Do

**Affiliations:** a Department of Physical Therapy, Graduate School of Sahmyook University; b Department of Physical Therapy, Gumi University, Seoul, Republic of Korea; c Department of Physical Therapy, School of Allied Health Professions, Loma Linda University, Loma Linda, CA, United States of America.

**Keywords:** electromyography, forward head posture, seat tilt, spinal curvature

## Abstract

Lumbar and pelvic alignment may have a huge impact on the posture of the spine and other parts. The aim of this study were to compare the spinal curvature of the cervical, thoracic, and lumbar spine and the muscle activity of the cervical erector spinae muscle, upper trapezius muscle, and thoracic erector spinae muscle when sitting at 3 different sloped, seating surfaces. A 10° wedge was used as the seating surface and we compared a forward sloping seat surface, a flat seating surface, and a rear sloping seat surface, in that order. Twenty healthy officers were recruited for this study. The subjects sat on the seat of 3 different slopes and watched a total of 3 videos, 10 minutes each. The rest time was 10 minutes. Subjects were photographed while viewing videos and muscle activity was measured. There were significant differences in cervical, thoracic, lumbar curvatures, and muscle activity in the 3 different sitting positions according to seat tilt (*P* < .05). Among the 3 slopes, the forward slope decreased forward head posture and cervical erector spinae muscle activity (*P* < .05). The activity of the cervical erector spinae muscle was 2.67% with a forward sloping seat, 5.45% with a flat sloping seat, and 6.77% with a rear sloping seat, revealing a significant difference (*P* < .05). This suggests that a forward sloping seat surface was effective in maintaining a neutral alignment of the spine, and this decreased the cervical spine erector muscle activity. Based on this result, equipment and chair development to incline seats forward may improve posture and health, and prevent chronic pain.

## 1 Introduction

As the world changes during the information age, the use of the video display terminals is increasing rapidly, which is also accompanied by a higher incidence of musculoskeletal disorders.^[1]^ Modern men tend to look at computers or smart media for prolonged periods of time, usually sitting, which causes changes in posture due to the influence of gravity. The most typical change in posture is the forward head posture (FHP).^[2]^ FHP has been reported to be due to a forward movement of the lower cervical spine and an increase in the upper cervical extensors activity, and it is a major cause of cervical spine dysfunction.^[3,4]^ This causes weakening of the upper trapezius, sternocleidomastoid, and pectoralis major muscles, and leads to disorders such as upper cross syndrome. The abnormal change in the joint and axial direction is compensated for by the upper trapezius, levator scapular, and supraspinatus. This will increased the activity of the muscles and cause early degeneration.^[5]^ This posture causes the peripheral section of the muscle fiber to shorten and to pull on the cervical joints, leading to chronic pain.^[6]^

Various approaches have been used for the treatment of the FHP, such as taping, proprioceptive exercise, stretching exercises, joint mobilization, endurance training, and deep neck flexor muscle strengthening exercises.^[7–9]^ However, although these treatments are effective, without fundamental changes in the patients posture and lifestyle, a complete recovery cannot be achieved.

The musculoskeletal system does not work independently, and each part has a direct effect on others.^[10]^ Lumbar and pelvic posture has a huge impact on the posture of the spine and other areas. Teaching subjects to use correct posture when sitting has a therapeutic effect on the prevention and treatment of problems in the spine. Various studies have investigated the effect of lumbar posture changes on cervical posture. Black et al (1996) studied the effects of multiple sitting postures on various spinal angles. When a slouched posture was compared to an erect sitting posture, slouched sitting posture was found to reduce lumbar lordosis and cervical lordosis, with increased FHP. The erect sitting position increases lumbar lordosis and influences the proper alignment of the cervical spine.^[11]^ When the erect sitting posture was compared to slouched sitting posture, the activity of the cervical erector spinae muscle decreased in the erect sitting posture as compared to the slouched sitting position.^[12]^ The results of such studies suggest that the correct alignment of the lumbar spine reduces the burden on the cervical vertebrae by reducing a FHP.

Many studies have been conducted to determine the ideal sitting position. According to the study of Frey and Tecklin (1986), the slope of the seat helps maintain lumbar lordosis. A forward sloping chair seat maintained a lordotic lumbar posture, similar to that seen in a standing position, better than the typical flat chair.^[13]^ Other studies found that a flat seat and a rear sloping seat tended to reduce the lumbar lordosis, while the forward sloping seat maintained the spine in lordosis.^[14,15]^

Most studies investigated effect of prolonged sitting on a forward sloping seat on the posture of the lumbar spine. However, there have not been any studies on the effect of a forward sloping seat on the alignment of the cervical spine posture or muscle activity. Therefore, we aim to compare the spinal curvature of the cervical, thoracic, and lumbar spine and the muscle activity of the cervical erector spinae muscle (CES), upper trapezius muscle (UT), and thoracic erector spinae muscle (TES) when sitting on 3 different sloped surfaces.

## 2 Method

### 2.1 Participants

Twenty asymptomatic office workers who work 8 hours a day and 40 hours a week participated in this study. Those who had malignancy, neurological, or musculoskeletal impairments diagnosed in the prior 6 months; those who had been treated due to pain in the neck, shoulder, or lumbar area; those who had a limited mobility of the spine during standing or bending; had pain in the temples; or had a history of illness or surgery. A confidence interval of 95%, a power of 80%, a mean effect size of 0.3 and alpha error probability of 0.05 were considered. Accordingly, the minimum sample size was calculated to be 20 persons using the G-power (ver. 3.1) software.

We described the purpose and process of the research to the subjects, and informed consent was obtained. This study was conducted with the approval of the Experimental Process Research Ethics committee of the Sahmyook University.

### 2.2 Procedures

This cross-over study measured the spinal curvature and muscle activity in the sitting position using 3 different slopes of the seating surface. After the subjects were educated about the research procedure prior to the experiment, we performed tests on muscle activity and spinal curvature.

The subjects feet were in contact with the ground holding the hip and the knee at 90°. If necessary, a footrest was used to maintain the angle of the hip and knee. The viewing monitor with a 24-inch display was placed in line with the center of the eye level of the subject. The distance between the eye and the monitor was set to 70 cm. The camera was set at a height of 0.93m using a tripod that was 1.5 m from the subject. We compared a 10°forward sloping surface, a flat surface and a 10°rear sloping surface. Between each trial, to prevent fatigue, the subject had a break of 10 minutes. Subjects were randomly measured in the prescribed seat tilt order.

### 2.3 Spinal curvature analyses

**Figure 1:**
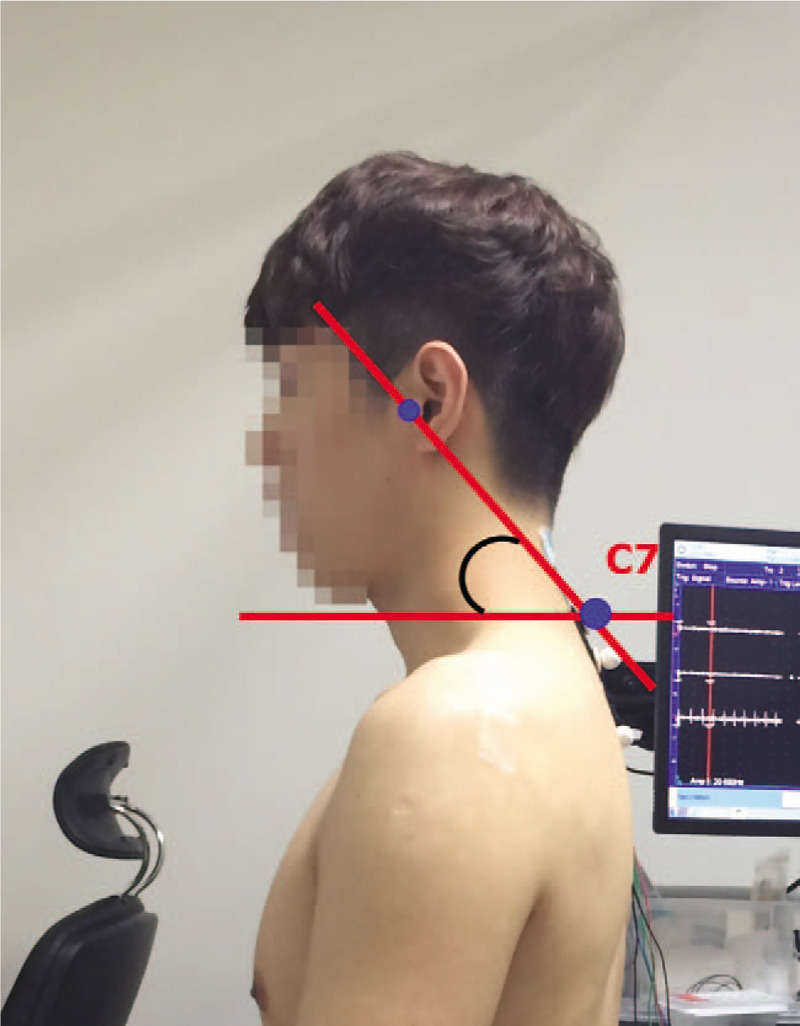
Craniovertebral angle.

**Figure 2:**
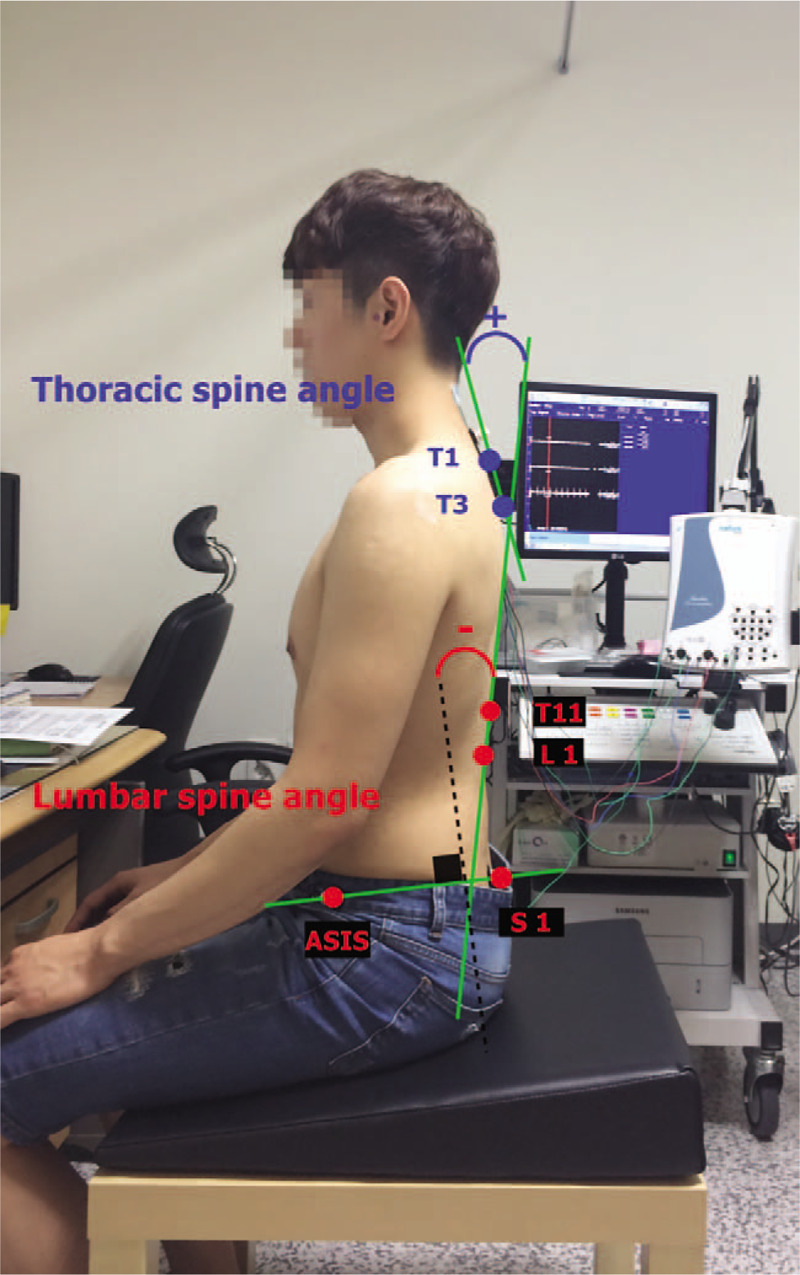
Maker placement and angle definition.

Spinal curvature angle was measured using an image process based on a Java program (Image J, ver. 1.32, National Institutes of Health). The craniovertebral angle was measured at the intersection of a horizontal line passing through the C7 spinous process and a line joining the midpoint of the tragus of the ear to the skin overlying the C7 spinous process (Fig. 1).^[16–18]^ The thoracic curvature angle was measured as the angle formed by 2 lines. Then, 2 lines connecting the 1^st^ and 3^rd^ thoracic spinous process and the 11^th^ thoracic and 1^st^ lumbar spinous process were drawn. The lumbar curvature angle was measured by the angle between the vertical lines. Finally, a line connecting from eleventh thoracic spinous process to first lumbar spinous process and the line connected from the anterior superior iliac spine to posterior superior iliac spine was drawn (Fig. 2).^[19]^

### 2.4 Electromyographic analyses

Before the measurement, the skin was shaved and washed with alcohol. For the CES, the electrodes were attached to the neck 1 cm away from the 4^th^ cervical spinous process; for the UT, they were attached to the middle point between the 7^th^ cervical spinous process and the acromion; for the TES, 3 cm away from the 4^th^ thoracic spinous process.^[12]^ Maximal voluntary contractions (MVC) are commonly used to obtain a reference amplitude because they then allow for signals to be expressed as a percentage of the assumed maximal muscle activity of the MVC (%). The maximum muscle contractions of each muscle were measured by the frequency and resistance previously reported.^[20]^ For the purpose of EMG data normalization, a series of 5 second MVC of the above mentioned muscles was performed prior to the sitting trial. Full details of these normalization procedures have been outlined previously.^[21]^

The activity of the CES, UT, and TES were measured by EMG (Nicolet EDX System Features Natus Medical). The sampling rate was set to 1000 Hz and the frequency bandwidth was set at 10 to 450 Hz. An electrocardiogram was used as a filter to minimize the influence of the electric signals of the heart rate. EMG signals measured from the muscles were calculated by taking the root mean square (RMS) value that provides a value close to the actual output value of the EMG signal after rectification. Each muscle's EMG was obtained and used as an average of the measured values normalized as a percentage using the MVC.

### 2.5 Statistical analysis

All of the statistics and the means and standard deviations were calculated using SPSS ver. 16.0. The data was confirmed for normal distribution using the Shapiro–Wilk test. We compared the measures using 1 way repeated measure analysis of variance (ANOVA). The Bonferroni was used as a post-hoc test. Statistical significance was set at 0.05.

## 3 Result

Baseline characteristics of the patients are shown in Table 1. Craniovertebral angle (CVA) had a significant angle difference in post-test results of 49.79°, 42.09°, and 36.39° from the forward slope, flat slope, and rear slope, respectively (*P* < .05). Thoracic curvature angle had a significant difference among the 3 slopes, with post-test 26.35°, 30.14°, and 35.50° for forward slope, flat slope, and rear slope, respectively (*P* < .05). Lumbar curvature angles were significantly different, –17.90°, 1.76°, and 17.51° for forward slope, flat slope, and rear slope, respectively (*P* < .05) (Table 2).

**Table 1:**
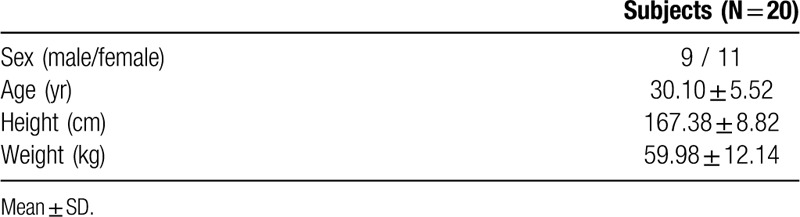
Baseline characteristics of the patients.

**Table 2:**
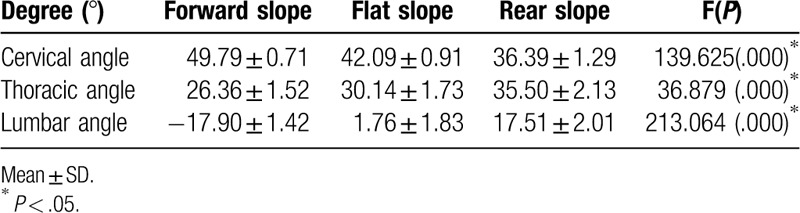
Comparison of spinal curvature angles in the 3 different seating surface.

The activity of the CES was 2.67% with a forward sloping seat, 5.45% with a flat sloping seat, and 6.77% with a rear sloping seat, revealing a significant difference (*P* < .05). There was no significant difference in the muscle activity of UT, with 2.22%, 3.27%, and 3.58% for forward slope, flat slope, and rear slope, respectively. The activity of the TES was not significantly different, with 5.80%, 6.91%, and 6.99% for forward slope, flat slope, and rear slope, respectively (Table 3).

**Table 3:**
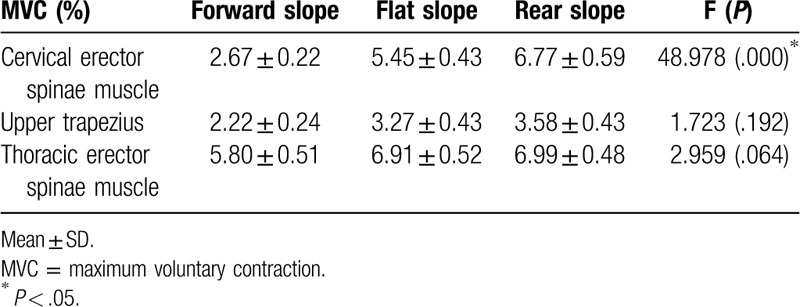
Comparison of muscle activity in the 3 different seating surface.

## 4 Discussion

Previous FHP studies have considered each body part separately without considering the overall interaction of the entire spinal column composed of the cervical, thoracic and lumbar spine. However, when viewed in terms of the chain theory, the spine is an organically linked relationship.^[10]^ Changes in the lumbar alignment can be a more effective way of changing the posture of the thoracic and cervical. Therefore, in this study attempted to change the posture of the whole spine by varying the alignment of the lumbar spine with various seating slopes.

Lumbar lordotic angles were decreased and thoracic kyphotic angles were increased more with rear sloping seat than with a flat and forward sloping seat. The CVA was decreased with rear sloping seat than a flat and forward sloping seat. The CVA is one of the simple and effective methods to evaluate FHP. In previous studies comparing 3 test methods to measure FHP, the CVA method was most relevant for measuring FHP. As the CVA decreases, the FHP increases.^[17]^ In this study, we found that the FHP was the most pronounced with a rear sloping seat. In a study by Caneiro et al 2010, the angle of the spine was measured in 3 sitting postures. This study positioned into 3 thoraco-lumbar sitting postures as defined by

1. lumbo-pelvic posture is anterior rotation of the pelvis in order to achieve a neutral lordosis of the lumbar spine and relaxation of the thorax;
2. Thoracic upright posture is anterior rotation of the pelvis, thoraco-lumbar spine extended and with shoulder blades slightly retracted;
3. Slump posture is posterior rotation of the pelvis, thoraco-lumbar spine relaxed while looking straight ahead. The FHP increased as the lordosis angle of the lumbar spine decreased.^[12]^ In the rear slope, the muscle activity of the CES was the highest at 6.77%, as compared to 2.67% in the forward slope, and 5.45% in the flat slope. This phenomenon causes a decrease in the lumbar lordosis angle due to the rear slope of the sitting surface and an increase in the FHP with increasing thoracic kyphosis. As the head moves forward, the external moment arm of the head / neck increases and would requires more muscle activity to support the head weight.^[12]^

The CVA was the greatest in the forward slope of the sitting surface, on which subjects demonstrated the least FHP and was close to the neutral alignment posture. In the forward slope, lumbar lordosis angle increased the most, and thoracic kyphosis angle was the most reduced. The activity of the CES was the lowest when compared to the other slopes, and it was found that neck loading was the least when the sitting surface was sloped forward.^[11,12]^ While sitting in a forward sloped seat, lumbar lordosis angles increased and thoracic kyphosis angle decreased, resulting in a relatively upright posture. These changes may affect the posture of head and neck and maintain a neutral posture, which may have affected the decreased CES activity.

In this study, there were no significant differences in UT and TES activity between each testing position. Caneiro et al (2010) compared cervico-thoracic muscle activity in 3 sitting positions

1. lumbar-pelvic sitting,
2. slump sitting, and
3. thoracic upright sitting. And found no significant difference in the activity of the UT.^[12]^ Szeto et al (2009) compared cervico-thoracic muscle activity in 2 resting positions for female office workers with neck pain and asymptomatic subjects. In the group with neck pain, UT activity increased at the position when the hand resting on the keyboard. In contrast, there was no difference in UT activity between the 2 resting positions in the asymptomatic group.^[22]^ Burnett et al (2009) measured the muscle activity of the UT during flexion and extension movements of the cervical spine in a lumbar-pelvic sitting position, but there was no significant difference.^[23]^ Previous studies have shown that the UT does not have a significant influence on maintaining the cervico-thoracic sitting posture or head movement.

Previous studies have compared thoracic erector spinae muscle activation in different positions. In a study by Sullivan et al (2002), the TES activity was measured in 20 healthy officers sitting in an upright position and slump position. The upright position showed higher thoracic erector spinae muscle activity than the slump position.^[24]^ O'Sullivan et al (2006) compared trunk muscle activity of normal subjects in an upright thoracic position, lumbo-pelvic position and slump position. In the upright thoracic position, the muscle activity of the TES was the highest, and there was no difference between the lumbo-pelvic position and the slump position.^[25]^ Previous studies comparing TES activity with different postures showed higher levels of muscle activity in the upright posture, possibly due to maintaining a specific upright posture. In this study, it was speculated that there was no difference in TES activity because it was measured in a natural seated position according to the slope of the seating surface.

This study showed that lumbar lordosis was increased, thoracic kyphosis was decreased, and the CVA was increased when sitting on a 10° forward sloping seat compared to other slopes. The activity of the CES was also decreased. A forward sloping seating surface was effective in maintaining the neutral position of the spine and reducing the FHP.

Much effort has been made to reduce the burden on office workers working in a sitting position. Many studies have been conducted to reduce load on the spine by changing the height of the chair, the shape of the armrest and the inclination of the backrest. These methods have limitation due to needing to change the chair itself. However, our findings suggest that utilizing simple wedge cushions may be effective in reducing load on the cervical spine in office worker.

There were some limitations of the study which need to be acknowledged. First, similar to previous postural studies, this study involved a small sample of participants which reduces the statistical power of the finding. Secondly, this study investigated subjects over a short period of time (10 minutes), it is not known whether similar results would be detected over a long period of time. All recordings were taken during a viewing task in a laboratory environment, while other usual situation such as typing on a computer may have yielded to some different results. Further studies are required to investigate situations that are similar to the actual office work for longer periods.

## 5 Conclusions

This study was performed to investigate the muscle activity and spinal curvature in 3 different sloped seating surfaces among 20 healthy officers. Of the 3 slopes, the forward slope showed the greatest increase in the lumbar lordosis angle and decreased thoracic kyphosis angle and FHP. A forward slope of the seat surface was effective in maintaining a neutral spinal alignment, with reduced CES activity. The results of this study suggest that equipment and chair development to incline seats may improve posture.

## Author contributions

**Conceptualization:** JongEun Yim, KwangSun Do.

**Data curation:** KwangSun Do, Junhyuck Park.

**Formal analysis:** JongEun Yim, Junhyunk Park.

**Investigation:** KwangSun Do.

**Methodology:** Everett Lohman.

**Supervision:** JongEun Yim.

**Writing – original draft:** KwangSun Do.

**Writing – review and editing:** JongEun Yim, Everett Lohman.
